# *Bartonella henselae* bacteremia in a mother and son potentially associated with tick exposure

**DOI:** 10.1186/1756-3305-6-101

**Published:** 2013-04-15

**Authors:** Ricardo G Maggi, Marna Ericson, Patricia E Mascarelli, Julie M Bradley, Edward B Breitschwerdt

**Affiliations:** 1 Intracellular Pathogens Research Laboratory, Center for Comparative Medicine and Translational Research, College of Veterinary Medicine, North Carolina State University, Raleigh, NC, USA; 2 Cutaneous Imaging Center, Department of Dermatology and Center for Drug Design, University of Minnesota, Minnesota, USA; 3 Galaxy Diagnostics Inc., Research Triangle Park, Durham, USA

**Keywords:** Bartonella, BAPGM, Bacteremia, Striae, Neuropathy, Neurological disorder

## Abstract

**Background:**

*Bartonella henselae* is a zoonotic, alpha Proteobacterium, historically associated with cat scratch disease (CSD), but more recently associated with persistent bacteremia, fever of unknown origin, arthritic and neurological disorders, and bacillary angiomatosis, and peliosis hepatis in immunocompromised patients. A family from the Netherlands contacted our laboratory requesting to be included in a research study (NCSU-IRB#1960), designed to characterize *Bartonella* spp. bacteremia in people with extensive arthropod or animal exposure. All four family members had been exposed to tick bites in Zeeland, southwestern Netherlands. The mother and son were exhibiting symptoms including fatigue, headaches, memory loss, disorientation, peripheral neuropathic pain, striae (son only), and loss of coordination, whereas the father and daughter were healthy.

**Methods:**

Each family member was tested for serological evidence of *Bartonella* exposure using *B. vinsonii* subsp. *berkhoffii* genotypes I-III, *B. henselae* and *B. koehlerae* indirect fluorescent antibody assays and for bacteremia using the BAPGM enrichment blood culture platform.

**Results:**

The mother was seroreactive to multiple *Bartonella* spp. antigens and bacteremia was confirmed by PCR amplification of *B. henselae* DNA from blood, and from a BAPGM blood agar plate subculture isolate. The son was not seroreactive to any *Bartonella* sp. antigen, but *B. henselae* DNA was amplified from several blood and serum samples, from BAPGM enrichment blood culture, and from a cutaneous striae biopsy. The father and daughter were seronegative to all *Bartonella* spp. antigens, and negative for Bartonella DNA amplification.

**Conclusions:**

Historically, persistent *B. henselae* bacteremia was not thought to occur in immunocompetent humans. To our knowledge, this study provides preliminary evidence supporting the possibility of persistent *B. henselae* bacteremia in immunocompetent persons from Europe. Cat or flea contact was considered an unlikely source of transmission and the mother, a physician, reported that clinical symptoms developed following tick exposure. To our knowledge, this is the first time that a *B. henselae* organism has been visualized in and amplified from a striae lesion. As the tick bites occurred three years prior to documentation of *B. henselae* bacteremia, the mode of transmission could not be determined.

## Background

Due to complex nutritional requirements and slow dividing times, which necessitate a prolonged incubation period for successful isolation, members of the genus *Bartonella* are highly fastidious microorganisms that are difficult to document microbiologically in patient blood or tissue specimens [1]. Over the past decade, evolving evidence supports the fact that these bacteria can induce long-lasting intravascular infections in animals that serve as reservoir-adapted hosts, whereas, more recently a number of *Bartonella* sp. have been associated with persistent bacteremia in immunocompetent human patients experiencing a spectrum of symptoms and somewhat diverse disease pathologies [2,3]. On a comparative medical basis, Bartonella infection induces similar pathology in dogs, people, and other animals [3,4]. Infection with one or more *Bartonella* sp. has also been associated with fever of unknown origin [5-9], and arthritic and neurological disorders [10-12].

In an effort to overcome limitations associated with the molecular detection and isolation of *Bartonella* spp. from animals and immunocompetent human patients, our laboratory developed a novel diagnostic platform based on biochemical optimization of a modified insect-cell culture liquid medium (*Bartonella* alpha Proteobacteria Growth Medium or BAPGM) [13]. The BAPGM platform consists of PCR amplification of *Bartonella* DNA from the patient’s extracted blood and serum samples before, and after BAPGM enrichment culture and from isolates, if obtained, following subculture of pre-enriched samples onto blood agar plates. Research utilization of this testing platform has facilitated the documentation of *B. henselae* bacteremia in patients from the Australia [14], England [14], and the United States [10-12,15]. In addition, utilization of the BAPGM enrichment blood culture platform has facilitated the isolation or molecular detection of other *Bartonella* spp. including *Bartonella vinsonii* subsp. *berkhoffii* genotypes I and II [10-12,15,16], *Bartonella koehlerae*[10,12,14,17], *Candidatus* Bartonella melophagi [18], and a novel *Bartonella* sp. related to *Bartonella volans*[19], from the blood of immunocompetent humans [10,11,13]. Standardized precautions are routinely used in our laboratory to avoid DNA contamination and negative controls are used in each step of the testing platform, including culture, DNA extraction and PCR negative controls. All conventional PCR amplicons are sequenced to confirm the *Bartonella* species and 16S-23S ITS strain type. In this study, four members of a family from the Netherlands were tested for serological, microbiological (isolation) or molecular (PCR amplification and DNA sequencing) evidence of *Bartonella* exposure or bacteremia, using five indirect fluorescent antibody assays and the *Bartonella* alpha Proteobacteria growth medium (BAPGM) enrichment culture platform, respectively. In addition, skin biopsies were obtained surgically from the son for BAPGM enrichment culture, histopathology, and laser confocal immunohistochemistry.

## Methods

### Patients and samples

While reviewing recently published literature relative to potential transmission of *Bartonella* species by ticks [20-24], the mother, a 58-year-old anesthesiologist from the Netherlands contacted one of the investigators in our laboratory (EBB) by email and requested BAPGM enrichment blood culture testing to determine if she and her son could have been infected with a *Bartonella* sp. following tick bites. It was ultimately decided that the family would be tested in conjunction with an ongoing research study designed to determine the prevalence of bacteremia in people with animal and arthropod exposure. This research study was reviewed and approved by the North Carolina State University Institutional Review Board (NCSU IRB#1960) to assure conformity with all confidentiality and patient assurance laws in the United States. In July 2008, prior to the onset of illness in the mother and son, all four family members had experienced tick bites at the family vacation home in Zeeland, located in southwestern Netherlands, where they reported a large deer population. Tick attachments had also occurred during subsequent summer vacations. The family did not own a cat or dog, and all family members denied exposure to cats and cat fleas. During the three years prior to testing in the Intracellular Pathogens Research Laboratory (IPRL), the mother reported fatigue, headaches, memory loss, irritability, disorientation, chest pain, syncope (two episodes), fine tremors, shoulder pain, joint pain, loss of coordination, and peripheral neuropathic pain involving the arms. The son (an 18-year-old student) reported an illness of three-year duration, with symptoms including: fatigue, headaches, fine tremors, red conjunctivae, cervical lymphadenopathy, and striae involving both legs and the buttock. In the context of this manuscript, striae are irregular bands, stripes or lines in the skin. Between July 2009 and July 2011, both the mother and son underwent multiple diagnostic evaluations for infectious and non-infectious diseases. Both had been treated for a potential *Borrelia* sp. infection with multiple antibiotic combinations (azithromycin, cefuroxime, metronidazole, rifampin, tetracycyline) for at least 6 weeks duration, on several occasions. The historical response to antibiotics was inconsistent and difficult to assess, but in general, symptoms would diminish while receiving antibiotics and reoccur weeks to months after antibiotic administration ended. The last date in which the mother and son took antibiotics was May 24, 2011 and July 7, 2011, respectively. The father (64-year-old investment banker) and daughter (20-year-old medical student) were not symptomatic prior to or at the time of sample collection. As a physician, the mother decided that shipping samples from Europe to the United States might compromise sample integrity, thereby contributing to false negative test results. Therefore, after flying to North Carolina, the mother and son’s blood samples were aseptically collected during the last week of July 2011, whereas the father and daughter’s samples were collected the first week of August 2011. After the two week sample collection period, the family flew back to the Netherlands to await research testing results, which required months to complete. Three aseptically collected blood and serum samples were obtained during a one-week period from the mother, father, son and daughter. Three skin biopsy samples, which included a blue nevus located over the right deltoid, and a striae lesion (0.4 × 0.3 cm, excised to a depth of 0.5 cm) located on the left thigh, were surgically obtained from the son at a hospital in Raleigh, North Carolina, during the same time frame as blood samples were being collected. Blood, serum and skin biopsy samples were processed and tested in the IPRL, as described below. Histopathological examination of hematoxylin and eosin stained tissue sections was performed by the consulting human pathologist for the local hospital. Frozen tissues were sent to the Cutaneous Imaging Center, Department of Dermatology and Center for Drug Design, University of Minnesota to determine if *Bartonella* organisms could be visualized.

### *Bartonella* spp. IFA testing

Antibody seroreactivity to *B. vinsonii* subsp. *berkhoffii* genotypes I, II and III, *B. henselae* (strain Houston I), and *B. koehlerae* were determined using previously described indirect fluorescent antibody tests [10,11,25].

### BAPGM enrichment culture

The BAPGM enrichment culture platform, which has been employed by several research laboratories [26-28] was used to assess for *Bartonella* sp. bacteremia. Briefly the procedure included inoculation of 2 ml of blood and up to 2 ml of serum collected the same day into a culture flask containing 10 ml of BAPGM. Cultures were incubated for up to 14 days at 36°C with 5% CO_2_ and 100% humidity with constant agitation. A milliliter of each enrichment culture sample was sub-inoculated onto blood agar plates (10% rabbit blood, TSA II) at 7 and 14 days after incubation of the BAPGM flask for potential colony formation. For each patient sample tested using the BAPGM platform, an un-inoculated BAPGM culture was processed in an identical and simultaneous manner to monitor for potential laboratory contamination (quality assurance).

### DNA extraction, PCR amplification and DNA sequencing

Using established assays, PCR targeting the *Bartonella* 16S-23S intergenic spacer (ITS) region was used to amplify *Bartonella* spp. DNA from extracted blood, serum, skin biopsies, BAPGM enrichment cultures and isolates [10]. All PCR products obtained after amplification of extracted DNA from blood, serum and tissue samples, after BAPGM enrichment, and from agar plate colonies were sequenced directly or after cloning. Bacterial species and strain were defined by comparing DNA sequence similarities with other sequenced bacteria deposited in the GenBank database using the Basic Local Alignment Search Tool (Blast version 2.0).

### *Bartonella* confocal microscopy

An excised skin biopsy sample from the son (striae lesion) was drop-fixed in formalin and stored at room temperature. As a negative control, a scalp skin 4-mm punch biopsy was drop-fixed in Zamboni’s fixative (0.03% (w/v) picric acid and 2% (w/v) paraformaldehyde) for 48 h at 4°C and then transferred to a 20% sucrose solution with 0.05% sodium azide in PBS for storage. Processing and multi-staining of tissues specimens was performed according to a previously published procedure [29,30]. Vertical sections, 60-microns thick, were mounted in and cut on a cryostat. Floating sections were then incubated with primary antibodies to Collagen Type IV at a 1:200 dilution (Southern Biotech, 1340–01), donkey anti-goat Cy3 at 1:500 dilution (Jackson Immunoresearch, West Grove, PA), and a mouse antibody to *Bartonella henselae* at a 1:100 dilution (Abcam, ab704-250) plus donkey anti-mouse Cy5 at a 1:500 dilution (Jackson Immunoresearch). Washed samples were subsequently fixed to cover slips in agar, dehydrated in ethanol, cleared with methyl salicylate, and mounted in DEPEX (Electron Microscopy Sciences, Hatfield, PA).

## Results

The son was not seroreactive to *B. henselae, B. koehlerae* or *B. vinsonii* subsp. *berkhoffii* genotype I, II or III antigens by IFA testing (Table 1). However, *B. henselae* (16S-23S SA2 strain type) DNA was amplified and successfully sequenced from his blood, serum, a BAPGM enrichment blood culture, and from the striae biopsy sample (Table 2). *Bartonella* spp. DNA was not amplified from the biopsy of the son’s blue nevus. In contrast to her son, the mother was seroreactive to *B. henselae* (at an endpoint titer of 1:64), *B. koehlerae* (1:64), and *B. vinsonii* subsp. *berkhoffii* genotypes II and III antigens (both 1:64), but was not seroreactive to *B. vinsonii* subsp. *berkhoffii* genotype I. *Bartonella henselae* SA2 bacteremia was confirmed in the mother by PCR amplification and DNA sequencing (460/460 bp homology with GenBank AF369529) of 16S-23S ITS PCR amplicons from an extracted blood sample and from colonies obtained on blood agar plates following BAPGM enrichment [10,11,18] from the same collection date. Isolation of *B. henselae* from the mother’s blood and amplification of *B. henselae* DNA after enrichment blood culture from the son supported active infection with viable intravascular bacteria. The father and daughter were not seroreactive to the *Bartonella* spp. test antigens used in this study and *B. henselae* DNA was not amplified from any of their blood, serum or BAPGM enrichment blood cultures. As detailed in previous studies [10,11], *Bartonella* DNA was not amplified from any of the simultaneously processed BAPGM negative culture controls (results not shown).

**Table 1:** Indirect fluorescent serum antibody results for the four family members

**Patient**	**Collection date**	***Bvb *TI**	***Bvb *TII**	***Bvb *TIII**	***Bh***	***Bk***
Son	7/25/2011	<16	<16	<16	<16	<16
	7/27/2011	<16	<16	<16	<16	<16
	7/29/2011	<16	<16	<16	<16	<16
Mother	7/25/2011	<16	64	<16	64	64
	7/27/2011	<16	64	64	64	64
	7/29/2011	<16	64	<16	64	64
Daughter	8/1/2011	<16	<16	<16	32	<16
	8/2/2011	<16	<16	<16	32	<16
	8/5/2011	<16	<16	<16	32	<16
Father	8/1/2011	<16	<16	<16	32	<16
	8/2/2011	<16	<16	<16	32	<16
	8/5/2011	<16	<16	<16	32	<16

*Bh*: *B. henselae*; *Bk*: *B. koehlerae*; *Bvb*: *B*. *vinsonii* subsp. *berkhoffii*.

TI,TII, TIII denote *B. vinsonii* subsp. *berkhoffii* genotypes I, II and III, respectively.

**Table 2:** ***Bartonella *spp. blood, serum, BAPGM enrichment blood culture, subculture and tissue PCR testing results for the four family members**

**Patient**	**Sample type**	**Collection date**	**PCR sample**	**PCR culture**	**PCR isolate**
Son	Serum	7/29/2011	Neg	Neg	Neg
	Blood	7/29/2011	Neg		
	Blue nevus	7/25/2011	Neg	Neg	Neg
	Normal skin	7/25/2011	Neg	Neg	Neg
	Striae	7/25/2011	*Bh*SA2	Neg	Neg
	Serum	7/25/2011	*Bh*SA2	Neg	Neg
	Blood	7/25/2011	*Bh*SA2		
	Serum	7/27/2011	*Bh*SA2	*Bh* SA2	Neg
	Blood	7/27/2011	*Bh*SA2		
Mother	Serum	7/25/2011	Neg	Neg	Neg
	Blood	7/25/2011	Neg		
	Serum	7/27/2011	Neg	Neg	*Bh* SA2
	Blood	7/27/2011	*Bh*SA2		
	Serum	7/29/2011	Neg	Neg	Neg
	Blood	7/29/2011	Neg		
Daughter	Serum	8/1/2011	Neg	Neg	Neg
	Blood	8/1/2011	Neg		
	Serum	8/2/2011	Neg	Neg	Neg
	Blood	8/2/2011	Neg		
	Serum	8/5/2011	Neg	Neg	Neg
	Blood	8/5/2011	Neg		
Father	Serum	8/1/2011	Neg	Neg	Neg
	Blood	8/1/2011	Neg		
	Serum	8/2/2011	Neg	Neg	Neg
	Blood	8/2/2011	Neg		
	Serum	8/5/2011	Neg	Neg	Neg
	Blood	8/5/2011	Neg		

**Figure 1:**
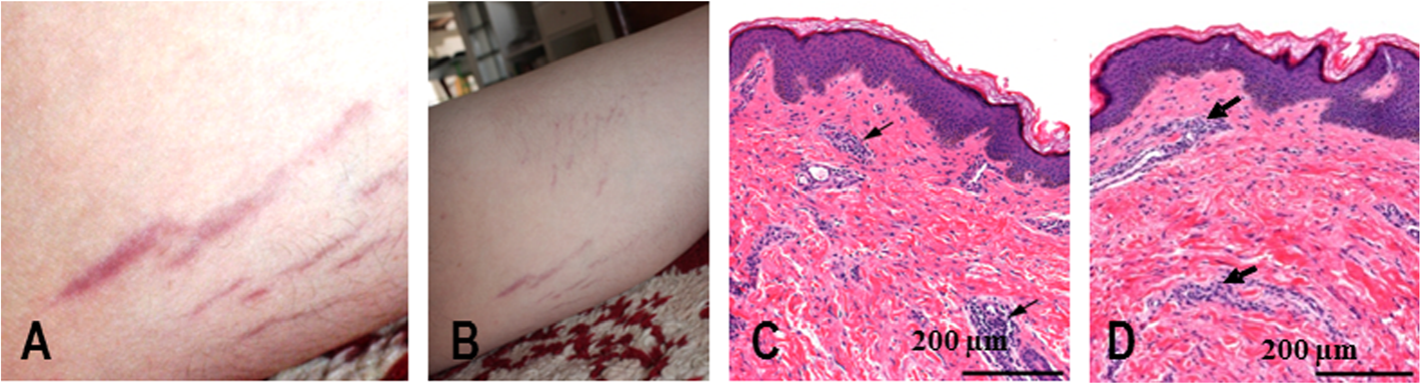
Gross appearance of striae located on the thigh of the son (A & B, photographs provided by the family) and the hematoxylin/eosin stained striae tissue biopsy (C & D) illustrating (arrows) minimal nonspecific superficial perivascular chronic inflammation.

**Figure 2:**
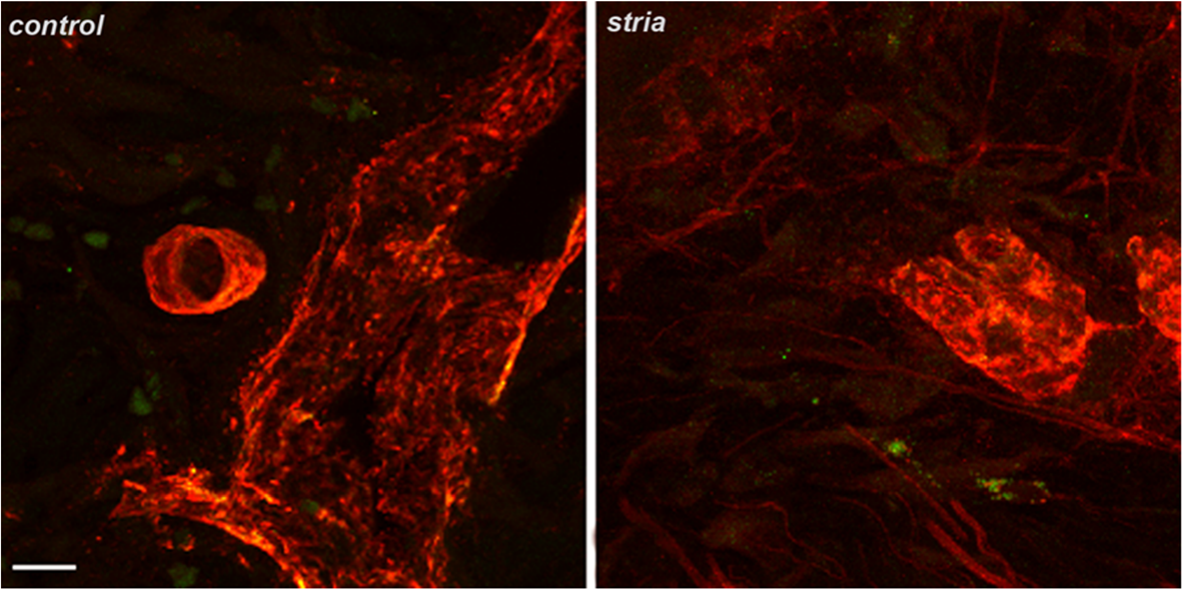
**Photomicrographs captured using laser scanning confocal microscopy demonstrating immunoreactive *B. henselae *organisms in the striae skin biopsy obtained from the son’s thigh (right panel, small green particles).** Left panel is scalp skin from a non-infected subject also immunostained with *B. henselae* antibody. No bacteria were visualized in control sections, although relatively large auto fluorescent red blood cells are partially visible (pseudo-colored green). Both samples also stained with collagen IV to highlight immunoreactive vascular tissue (pseudo-colored red). Note bacteria within skin appear external to vascular tissues. Images are projections of 31, 0.48-micron thick optical sections, total thickness 15 microns. Scale bar = 10 microns.

The gross appearance of the son’s striae are depicted in Figure 1A and 1B. Histopathology of the right deltoid skin lesion contained features of a blue nevus. The striae lesional biopsy from the left thigh contained minimal nonspecific superficial perivascular chronic inflammation (Figure 1C and 1D). Immunostaining of the striae tissue from the son’s skin biopsy revealed *B. henselae* immuno-positive staining within the dermis and by confocal microscopy imaging, the bacteria were external to vascular tissue (Figure 2).

After these results were obtained, the son elected to not be treated with additional antibiotics. During 2012 he remained mostly healthy, and passed Propedeuse exam Cum Laude. The striae regressed spontaneously leaving slight, colorless and painless marks. Conjunctivitis and the fine motor tremor have persisted. Psoriasis was diagnosed in 2012 for which he is being treated with vitamin supplementation and immune stimulants. The mother also did not seek treatment for *B. henselae* bacteremia until several months after test results became available, when she developed severe myalgia, tendinitis involving several joints and paresthesias. She was treated with intraveneous ceftriaxone 4 gram twice a week for six months. After three months of antibiotic therapy, Bartonella serology was reported as positive (IFA titer 1:32—normal < 32). During the final two months of antibiotic therapy, rifampin, hyperthermic therapy every day (sauna) and hyperbaric oxygen therapy by mask in a high-pressure cabin 2.4 bar, for 75 minutes each day were administered for 45 days. None of these therapies decreased the woman’s symptoms. As of March 2013, the father and daughter have remained healthy.

## Discussion

In this study, *B. henselae* bacteremia was confirmed in the two symptomatic family members, but not in the two historically healthy family members. Although the symptoms that developed in 2008 as reported by the physician and her son were similar to questionnaire respondent symptoms reported by other patients with *B. henselae* bacteremia [10,11], it is not possible to determine whether the symptoms in these two family members were due in part or total to infection with this organism*.* Unless re-infection was occurring, prior efforts to eliminate *B. henselae* with antibiotics were not successful, further supporting the possibility that antibiotic treatment failure can occur in a subset of *B. henselae* bacteremic patients [14,31]. As our research group was not involved in patient management decisions prior to or after *B. henselae* bacteremia was confirmed, the purpose of this report was to provide evidence supporting *B. henselae* bacteremia in two immmunocompetent individuals from Europe, who believed that tick transmission was the most likely source of their infections. During the previous three years, all four family members reported tick bites while vacationing in southwestern Holland and all denied exposure to cats. As *B. henselae* DNA was successfully amplified and sequenced from one of the son’s BAPGM enrichment blood cultures and from a subculture isolate obtained from the mother’s blood, bacteremia (viable bacteria) was confirmed in both individuals despite prior administration of antibiotics. Only one of three blood culture sample sets from the mother and the son resulted in enrichment culture or isolation evidence to support the presence of bacteremia, which is consistent with prior experience with the BAPGM platform when testing sequentially-obtained blood samples from sick human patients [10,11,16,17,19,32]. Presumably, failure to document infection in two of the three sample sets is potentially due to a relapsing pattern of bacteremia in humans [33-36], as has been reported in experimentally-infected cats [37,38] and rodents [33,39,40]. Due to diagnostic limitations associated with serology, direct blood plating methods, and PCR following DNA extraction directly from patient samples, our research group developed and have used a combined method that incorporates PCR from extracted blood, serum and BAPGM enrichment blood culture [10,11,17]. This study further supports the enhanced diagnostic sensitivity of the BAPGM enrichment blood culture platform for documentation of *Bartonella* spp. bacteremia in immunocompetent patients, and provides additional support for the need to test three sample sets obtained during a one week collection period [32].

In addition to documentation of bacteremia, immunohistochemical confocal microscopy was used to visualize *B. henselae* organisms in the son’s striae tissue biopsy. This observation provides preliminary evidence to support a potential association between *B. henselae* infection and striae. Although lay publications have frequently reported [41,42] that striae are caused by *Bartonella* sp. infections, to our knowledge there are no scientific publications that have investigated, reported, or confirmed this possibility. Clearly, additional immunohistochemical studies of striae would be of interest to determine if persistent *Bartonella* sp. bacteremia could contribute to the development of these skin lesions in human patients.

As is true throughout much of the world, *B. henselae* infection in immunocompetent people has been reported previously in the Netherlands in association with the diagnosis of typical or atypical cases of cat scratch disease (CSD) [43,44]. Cats, infected with *B. henselae* by cat fleas (*Ctenocephalides felis*), develop a bacteremia that can persist for years [37,45-49]. The term cat scratch disease is clearly of historical medical importance, but continued use of the term as a sole reference to *B. henselae* or other *Bartonella* sp. infections is potentially detrimental for patient diagnosis and patient management decisions. Briefly, *B. henselae,* the acknowledged cause of CSD, has also been documented (by PCR or isolation) in dogs [13,25,50-54], dolphins [55,56], feral swine [57], horses [58-60], and Beluga whales [61]. In North America, *B. henselae* is the most frequent *Bartonella* species isolated from bacteremic sick dogs [54] and people [10,11,62]. In a recent report of a patient with neuroretinitis in Australia, a well-documented ocular pathology induced by *B. henselae,* bartonellosis was diagnosed following the bite (sting) of a bull ant (genus *Myrmecia*) [21]. Those authors also advocated for medical use of the more inclusive term bartonellosis. Thus, because of alternative vectors, numerous accidental or reservoir hosts, and the seemingly broad spectrum of disease manifestations, referring to all *B. henselae* infections as CSD is a contradiction in fact, and the use of the more globally applicable term such as bartonellosis is suggested. In addition, CSD is considered a self-limiting infection for which antibiotic therapy is not recommended, whereas more recent evidence indicates that antibiotic elimination of *B. henselae* bacteremia can be extremely challenging, and potentially difficult to achieve [19,31]. Interestingly, molecular differences among *B. henselae* isolates based on 16S rDNA sequences and multiple-locus variable number tandem repeat analysis (MLVA), have documented the presence of two different *B. henselae* genotypes, one more frequently observed in association with human infections (genotype I), and the second (genotype II) most often isolated from bacteremic cats, suggesting that genotype II isolates may be minimally or nonpathogenic for humans (CSD) as compared with a more pathogenic and zoonotic genotype I [63,64].

In recent years, a substantial number of European studies have reported the presence of *B. henselae* DNA in ticks, including *I. ricinus*[24,65-76], suggesting that ticks may act as an important ecological reservoir for this *Bartonella* species. In addition, several recent publications have provided indirect [22,23] or experimental vector competence evidence [24] to support transmission of *Bartonella spp.* (including *B. henselae*, *B. birtlesii*, and *B. vinsonii* subsp. *berkhoffii*) by ticks (including *I. ricinus*) [22-24]. Two previous studies have not documented *Bartonella* spp. DNA in ticks from the Netherlands [46,77], where this family experienced tick exposures. Although not reported specifically in the results, *B. henselae* SA2 strain DNA was amplified and sequenced from two *I. ricinus* ticks from Klasdorf, Brandenburg, Germany, by targeting the 16S-23S rRNA intergenic spacer region [73] (Kempf VA, Maggi RG, unpublished data). The DNA sequences derived from these two ticks were identical to the *B. henselae* DNA sequences obtained from the two bacteremic people in this report. Interestingly, the remaining eight ticks that were sequenced as part of that study to confirm the *Bartonella* species and strain type contained a *B. henselae* 16S-23S Houston 1 DNA and were collected in southern Germany, France and Portugal, suggesting the possibility of *B. henselae* strain variation among ticks. It is also important to consider the tick life stage, as in that study the odds of detecting *B. henselae* DNA was 14 fold higher in nymphal ticks as compared to adult ticks [73]. In addition, differences in PCR sensitivity among studies and differences in *Bartonella* sp. gene targets used to test tick DNA extractions can contribute to divergent findings among laboratories, when testing ticks from the same location. Most previous studies designed to detect and identify *Bartonella* species in *I. ricinus* ticks from Europe have targeted the citrate synthase (*gltA*) and 16S rRNA genes. Unfortunately, these genes have very limited genetic variability and therefore are not optimal to differentiate among *B. henselae* strains. Clearly the role of ticks as potential vectors for transmission of *Bartonella* sp. to animals and humans deserves additional research consideration.

## Conclusion

Although *B. henselae* infection was documented by PCR amplification and DNA sequencing in both of the sick members of this family, there may or may not be a causal relationship between the bacteria and the reported symptoms. *Bartonella* serology was supportive of *B. henselae* infection in the mother, but not in the son, further supporting reports of seronegative *Bartonella* sp. bacteremia in human patients [10,11,78,79]. Also, despite improvements in PCR sensitivity, immunohistochemical methods are useful to facilitate the visualization of *Bartonella* species in patient tissue specimens. Finally, as ticks removed from the patients were not saved for PCR testing, it is not clear whether infection with *B. henselae* was acquired by tick bites or by another mode of transmission.

### Consent

Written informed consent was obtained from the patients for publication of this report and any accompanying images.

## Abbreviations

ITS: Intergenic spacer; BAPGM: *Bartonella* alpha proteobacteria growth medium; IPRL: Intracellular pathogens research laboratory.

## Competing interests

In conjunction with Dr. Sushama Sontakke and North Carolina State University, Dr. Breitschwerdt holds U.S. Patent No. 7,115,385; Media and Methods for cultivation of microorganisms, which was issued October 3, 2006. He is the chief scientific officer for Galaxy Diagnostics, a company that provides diagnostic testing for the detection of *Bartonella* species infection in animals and human patients. Dr. Ricardo Maggi has led research efforts to optimize the BAPGM platform and is the Scientific Technical Advisor for Galaxy Diagnostics. All other authors have no potential conflicts.

## Authors’ contributions

RM and PM performed the BAPGM enrichment blood culture and PCR testing of the patient samples, performed DNA sequencing and alignments, and generated the first draft of the manuscript. ME performed the immunohistochemistry and confocal microscopy. JB assisted in sample acquisition and serological testing. EB coordinated various aspects of the investigation and helped to draft the final manuscript. All authors read and approved the manuscript.
